# Investigation of COSMO-SAC model for solubility and cocrystal formation of pharmaceutical compounds

**DOI:** 10.1038/s41598-020-76986-3

**Published:** 2020-11-16

**Authors:** Samane Zarei Mahmoudabadi, Gholamreza Pazuki

**Affiliations:** Department of Chemical Engineering, Amirkabir University of Technology (Tehran Polytechnic), Tehran, Iran

**Keywords:** Biomedical engineering, Chemical engineering

## Abstract

In this study, a predictive model named COSMO-SAC was investigated in solid/liquid equilibria for pharmaceutical compounds. The examined properties were the solubility of drug in the pure and mixed solvents, octanol/water partition coefficient, and cocrystal formation. The results of the original COSMO-SAC model (COSMO-SAC (2002)) was compared with a semi-predictive model named Flory–Huggins model and a revised version of the COSMO-SAC (COSMO-SAC (2010)). The results indicated the acceptable accuracy of the COSMO-SAC (2002) in the considered scope. The results emphasized on the suitability of the COSMO-SAC model for simple molecules containing C, H, and O by covalent and hydrogen bonding interactions. Applicability of the COSMO-SAC for more complicated molecules made of various functional groups such as COO and COOH doubly requires more modification in the COSMO-SAC.

## Introduction

Knowing of phase equilibria, and thermodynamic properties such as solubility and partition coefficient for pharmaceutical compounds has wide applications in the design, development, and optimization of their manufacturing in laboratory or industry scale. Besides of experimental approach, which is time-consuming and expensive, the mathematical modeling gathered attentions due to lower cost and wide working range without further limitations from the substance type and ambient conditions. Generally, three aspects reported for the thermodynamic modeling are: (1) semi-empirical model, (2) semi-predictive model, (3) predictive model, which have different accuracies and reliable ranges. The theoretical quantum chemistry applied in the model proposal and the need to experimental data are the most significant differences between groups (2) and (3). While the semi-empirical models often are correlations without theoretical meaning obtained by experiment for certain species.

Among the mentioned models, the predictive models estimate entirely the desired properties by knowing only the molecular structure without the further requirement to experimental data. The UNIFAC^1^, the NRTL-SAC^2^, the COSMO-RS^3–5^, and the COSMO-SAC^6^ are a few examples. The predictive models, such as the UNIFAC, primarily defined based on functional group and several adjustable parameters. In contrast, other two predictive models, such as the COSMO-RS and the COSMO-SAC, are conductor-like screening models-realistic solvation and compute activity coefficient based on the computational quantum mechanics by knowing the molecular structure and fewer adjustable parameters in comparison to the UNIFAC. The COSMO-RS is the firstly developed by extension of a dielectric continuum-solvation model to liquid phase thermodynamics, and the COSMO-SAC is a modified version of the COSMO-RS^7^.

The several researcher studied the COSMO-SAC and the COSMO-RS. Tung et al*.*^8^ compared the NRTL-SAC and the COSMO-SAC to predict pharmaceutical solubilities for Lovastatin, Simvastatin, Rofecoxib, and Etoricoxib. Zhou et al*.*^9^ applied the COSMO-SAC to separate thioglycolic acid from its aqueous solution by ionic liquids. Paese et al*.*^10^ considered the COSMO-SAC for predicting phase equilibria of aqueous sugar solutions and industrial juices. Xavier et al*.*^11^ studied vapor–liquid equilibria (VLE) of systems containing fragrances using the COSMO-SAC. Bouillot et al*.*^12^ investigated drug solubilities by the COSMO-SAC. Shu and Lin^13^ predicted drug solubility in mixed solvent systems using the COSMO-SAC activity coefficient model. Buggert et al*.*^14^ applied the COSMO-RS for partition coefficient calculations. Hsieh et al*.*^15^ considered the original COSMO-SAC (COSMO-SAC 2002) and revised the COSMO-SAC models (COSMO-SAC 2010) for solubility and octanol/water partition coefficient for pharmaceutical compounds. They reported a 388% error for solubility prediction from the original COSMO-SAC (COSMO-SAC 2002).

In contrast to researchers focused on the predictive ability of the COSMO-SAC for different systems, some authors studied the primary quantum mechanism applied in the COSMO-SAC and developed various data bank. Mullins et al*.*^16^ developed a database consist of 1432 COSMO files and provided FORTRAN code for sigma profile and activity computations. Bell et al*.*^17^ assembled an extensive database of COSMO files for 2261 compounds. Ferrarini et al*.*^18^ distributed a sigma-profile database for a wide range of molecules using the GAMESS software. They also tested different quantum chemistry theories for the calculation of the electronic structure. Mu et al*.*^19^ examined the performance of COSMO-RS with sigma profiles from different theories.

Some authors modified the COSMO-SAC model in order to increase accuracy. Lee and Lin^20^ added Peng–Robinson EOS to the COSMO-SAC. Firstly, Lin et al*.*^21^ introduced the concept of modifying sigma profile to enhance model precisions. Hsieh et al*.*^22^ improved the COSMO-SAC for vapor–liquid and liquid–liquid equilibrium calculations by separating the sigma profile into HB-OH, HB-nonOH, and non-HB. Afterward, Paulechka et al*.*^23^ revised the COSMO-SAC model by splitting the sigma profile into OH and non-OH parts and Islam and Chen^24^ proposed a method for the sigma profile generation input into the COSMO-SAC.

The object of this study is to investigate the performances of two existing predictive models based on COSMO calculations, the COSMO-SAC (2002) and the COSMO-SAC (2010), for pharmaceutical compounds and to compare it with another widely applicable predictive model called the Flory–Huggins model^25^. By comparison of the COSMO-SAC to another predictive model such as the Flory–Huggins model, its unremarkable impacts in the predictive model scope is determined. The examined pharmaceutical compounds contain H, C, O, N, S, F, and Cl atoms and include at least one hydrogen bonding or double bond between atoms. The solubility in binary and ternary systems, octanol/water partition coefficient, and cocrystal formation are of interest in the current study. For solubility in the binary system, 918 data for 110 systems for 35 pharmaceutical compounds are over temperature ranges 262–360 K and the mole fractions $1 \times 10^{ - 7}$ to 0.7. Afterward, two systems of cocrystal formation, sulfamethazine-salicylic acid in methanol solvent and carbamazepine-acetyl salicylic acid in ethanol, are investigated by the COSMO-SAC (2002) model which have not been studied before.

## Methods

### COSMO file and sigma profile

As described before, the basis of the COSMO-SAC model is quantum mechanics through density function theory calculations. Several commercial or free software provide preliminary information for COSMO-SAC in the form of a text file called COSMO-file. Dmol^3^ module in Materials Studio and academic free software GAMESS are few examples. In COSMO calculations, a molecule separates into several parts called segment and charge distributions over entire segments are calculated in order to neutralize whole molecule. Location of segments, segment areas and charge densities are the computed properties in COSMO file. In order to perform COSMO-SAC calculations, the following data must obtain from COSMO-file: (1) surface area ($A$) and cavity volume of the molecule ($V$), (2) location of segment (a vector with x, y and z coordination), its charge density ($\sigma_{n}^{ * }$) and area ($A_{n} (\sigma )$). The mentioned information were modified in order to make the sigma profile ($p(\sigma )$) required for COSMO-SAC calculations. Klamt et al*.*^4^ introduced the following equation to average the charge densities from COSMO-file

1 $$\sigma_{m} = \frac{{\sum\nolimits_{n} {\sigma_{n}^{ * } \frac{{r_{ave}^{2} r_{n}^{2} }}{{r_{ave}^{2} + r_{n}^{2} }}\exp \left( { - \frac{{d_{mn}^{2} }}{{r_{ave}^{2} + r_{n}^{2} }}} \right)} }}{{\sum\nolimits_{n} {\frac{{r_{ave}^{2} r_{n}^{2} }}{{r_{ave}^{2} + r_{n}^{2} }}\exp \left( { - \frac{{d_{mn}^{2} }}{{r_{ave}^{2} + r_{n}^{2} }}} \right)} }}$$

In the above equation, *d*_*mn*_ is the distance between two segments *n* and *m*. The *r*_*n*_ (segment radius) is obtained from segment area as follows:

2 $$r_{n} = \left( {\frac{{A_{n} }}{\pi }} \right)^{0.5}$$

Mullins et al*.*^16^ reported the value of r_ave_. The sigma profile defined as the probability of finding segments with charge density $\sigma_{m}$:

3 $$p(\sigma_{m} ) = \frac{{n(\sigma_{m} )}}{{\sum\nolimits_{m} {n(\sigma_{m} )} }} = \frac{{A(\sigma_{m} )}}{{\sum\nolimits_{m} {A(\sigma_{m} )} }}$$

where *n* is determined from accounting the number of segments with specific charge density $\sigma_{m}$ and $A(\sigma_{m} )$ is surface area with charge density $\sigma_{m}$.

Generally, for most molecules, charge density values range between − 0.025 to 0.025 $\frac{e}{{\dot{A}^{2} }}$. Four steps for generating the sigma profile are as below:

1. Consider 50 intervals by 0.001 increments in charge density range − 0.025 to 0. 025.
2. Each interval is defined by lower and upper bounds, $\sigma_{left}$ and $\sigma_{right}$. Firstly, find the charge densities distributed at interval *i* and calculated their contributions according to:
  4 $$w_{i} (\sigma ) = \frac{{\sigma - \sigma_{i,left} }}{0.001}$$
3. Afterward, calculate probabilities at lower and upper bounds of interval *i* as below:
  5 $$A(\sigma_{i,left} )p(\sigma_{i,left} ) = \sum\limits_{{\sigma_{i,left} }}^{{\sigma_{i,right} }} {w_{i} (\sigma )A(\sigma )}$$
  6 $$A(\sigma_{i,right} )p(\sigma_{i,right} ) = \sum\limits_{{\sigma_{i,left} }}^{{\sigma_{i,right} }} {[1 - w_{i} (\sigma )]A(\sigma )}$$
4. The sigma profile is generated by plotting sigma values versus the calculated probabilities.

As described in literature review, some authors divided the sigma profile into parts to have a better description of hydrogen-bonding (hb) interactions. Hsieh et al*.*^22^ proposed to separate the sigma profile into non hydrogen bounding, hydroxyl group (OH) and non-hydroxyl group as follows equation (COSMO-SAC (2010)):

7 $$p(\sigma_{m} ) = p^{NHB} (\sigma_{m} ) + p^{OH} (\sigma_{m} ) + p^{OT} (\sigma_{m} )$$

where $p^{NHB} (\sigma_{m} )$ donates probabilities of all non-hydrogen bounding atoms, $p^{OH} (\sigma_{m} )$ shows probabilities of OH bounding and $p^{OT} (\sigma_{m} )$ determines F, N, and hydrogen atoms connected to F and N atoms. The above-mentioned contributions were determined as follows:

8 $$p^{OH} (\sigma_{m} ) = \frac{{A^{OH} (\sigma_{m} )}}{{A^{OH} (\sigma_{m} ) + A^{OT} (\sigma_{m} )}}p(\sigma_{m} )\left( {1 - \exp \left( { - \frac{{\sigma^{2} }}{{\sigma_{o}^{2} }}} \right)} \right)$$

9 $$p^{OT} (\sigma_{m} ) = \frac{{A^{OT} (\sigma_{m} )}}{{A^{OH} (\sigma_{m} ) + A^{OT} (\sigma_{m} )}}p(\sigma_{m} )\left( {1 - \exp \left( { - \frac{{\sigma^{2} }}{{\sigma_{o}^{2} }}} \right)} \right)$$

where $\sigma_{o}$ is threshold for hydrogen bounding determination and its values is 0.007 $\frac{e}{{\dot{A}^{2} }}$.

### COSMO-SAC model

#### COSMO-SAC (2002)

In the COSMO-SAC model, activity coefficients computed by solvation energy were obtained from ab initio solvation calculation at two steps: (1) the dissolution of a solute in the conductor, (2) conversion of the conductor into a real solvent. The activity coefficient of component *i* in solvent *S* in the COSMO-SAC ($\gamma_{i,S}$) obtained by considering two contributions; combinatorial part.

($\gamma_{i,s}^{C}$) and residual part($\gamma_{i,s}^{R}$) as follows^6^:

10 $$\ln \gamma_{i,S} = \ln \gamma_{i,s}^{C} + \ln \gamma_{i,s}^{R}$$

The size and shape differences of the molecules are accounted in the combinatorial part and calculated by the Staverman–Guggenheim term as follows^26^:

11 $$\ln \gamma_{i,s}^{C} = \ln \frac{{\phi_{i} }}{{x_{i} }} + \frac{z}{2}q_{i} \ln \frac{{\theta_{i} }}{{\phi_{i} }} + l_{i} - \frac{{\phi_{i} }}{{x_{i} }}\sum\limits_{j} {x_{j} l_{j} }$$

where $\theta_{i}$, $\phi_{i}$ and $l_{i}$ are defined as follows:

12 $$\theta_{i} = \frac{{x_{i} q_{i} }}{{\sum\nolimits_{i} {x_{i} q_{i} } }};\phi_{i} = \frac{{x_{i} r_{i} }}{{\sum\limits_{i} {x_{i} r_{i} } }};l_{i} = \frac{z}{2}\left( {r_{i} - q_{i} } \right) - (r_{i} - {1})$$

In the above expressions, $q_{i}$ and $r_{i}$ are related to cavity volume of component *i* ($V_{i}$) and total surface area of molecule *i* ($A_{i}$) obtained from the COSMO-file and defined as follows:

13 $$r_{i} = \frac{{V_{i} }}{{r_{o} }};q_{i} = \frac{{A_{i} }}{{q_{o} }}$$

where $r_{o}$ and $q_{o}$ are the normalized volume and normalized surface area. The residual part of the COSMO-SAC (2002) was defined as follows^6,17^:

14 $$\ln \gamma_{i,s}^{R} = n_{i} \sum\limits_{{\sigma_{m} }} {p_{i} (\sigma_{m} )\left[ {\ln (\Gamma_{S} (\sigma_{m} )) - \ln (\Gamma_{i} (\sigma_{m} ))} \right]}$$

where $n_{i}$, effective segment number of molecule *i*, is correlated with effective segment surface area ($a_{eff}$) and surface area of molecule *i* ($A_{i}$) according to below expression:

15 $$n_{i} = \frac{{A_{i} }}{{a_{eff} }}$$

where $\Gamma (\sigma_{m} )$ is the segment activity coefficient and calculated from:

16 $$\ln (\Gamma_{S} (\sigma_{m} )) = - \ln \left\{ {\sum\limits_{{\sigma_{n} }} {p_{S} (\sigma_{n} )\Gamma_{S} (\sigma_{n} )\exp \left[ { - \frac{{\Delta W(\sigma_{m} ,\sigma_{n} )}}{RT}} \right]} } \right\}$$

17 $$\ln (\Gamma_{i} (\sigma_{m} )) = - \ln \left\{ {\sum\limits_{{\sigma_{n} }} {p_{i} (\sigma_{n} )\Gamma_{i} (\sigma_{n} )\exp \left[ { - \frac{{\Delta W(\sigma_{m} ,\sigma_{n} )}}{RT}} \right]} } \right\}$$

The exchange energy $\Delta W(\sigma_{m} ,\sigma_{n} )$ is defined:

18 $$\Delta W(\sigma_{m} ,\sigma_{n} ) = \left( {\frac{{\alpha^{\prime}}}{2}} \right)\left( {\sigma_{m} + \sigma_{n} } \right)^{2} + c_{hb} \max \left[ {0,\sigma_{acc} - \sigma_{hb} } \right]\min \left[ {0,\sigma_{don} + \sigma_{hb} } \right]$$

The $c_{hb}$ and $\sigma_{hb}$ are the energy-type constant and cutoff value for hydrogen bonding interaction^16^. The $\sigma_{acc}$ and $\sigma_{don}$ are maximum and minimum values of $\sigma_{m}$ and $\sigma_{n}$. $\alpha^{\prime}$ accounts the misfit energy and the *T* and *R* are system temperature and the universal gas constant. The values of above mentioned parameters are reported in Mullins et al*.*^16^. In Eq. (16), the sigma profile for the mixture ($P_{S} (\sigma )$) are obtained from:

19 $$P_{S} (\sigma ) = \frac{{\sum\nolimits_{i} {x_{i} A_{i} (\sigma )P_{i} (\sigma )} }}{{\sum\nolimits_{i} {x_{i} A_{i} (\sigma )} }}.$$

#### COSMO-SAC (2010)

After establishing NHB, OH, and OT sigma profiles, the segment activity coefficient calculates as follows:

20 $$\ln \Gamma_{j}^{t} (\sigma_{m}^{t} ) = - ln\left[ {\sum\limits_{s}^{NHB,OH,OT} {\sum\limits_{{\sigma_{n} }}^{{}} {p^{s} \left( {\sigma_{n}^{s} } \right)\Gamma_{j}^{s} \left( {\sigma_{n}^{s} } \right)\exp \left( {\frac{{ - \Delta W\left( {\sigma_{m}^{t} ,\sigma_{n}^{s} } \right)}}{RT}} \right)} } } \right]$$

where subscript $j$ shows pure liquid or mixture and subscript $t$ denotes NHB, OH, and OT sites. The exchange energy has defined based on interaction between segments of different types, and is given by:

21 $$\Delta W(\sigma_{m} ,\sigma_{n} ) = \left( {A_{ES} + \frac{{B_{ES} }}{{T^{2} }}} \right)\left( {\sigma_{m} + \sigma_{n} } \right)^{2} + c_{hb} (\sigma_{m} ,\sigma_{n} )\left( {\sigma_{m} - \sigma_{n} } \right)^{2}$$

In contrast to COSMO-SAC (2002), the hydrogen bounding interaction *c*_*hb*_ has variable values for the contributions OH and OT:

22 $$_{hb} (\sigma_{m}^{t} ,\sigma_{n}^{s} ) = \left\{ {\begin{array}{*{20}l} {c_{OH - OH} } \hfill & {t = s = OH,\sigma_{m}^{t} .\sigma_{n}^{s} < 0} \hfill \\ {c_{OT - OT} } \hfill & {t = s = OT,\sigma_{m}^{t} .\sigma_{n}^{s} < 0} \hfill \\ {c_{OH - OT} } \hfill & {t = OH{,}s = OT,\sigma_{m}^{t} .\sigma_{n}^{s} < 0} \hfill \\ 0 \hfill & {otherwise} \hfill \\ \end{array} } \right.$$

Three hydrogen bounding interaction parameters (*c*_*OH-OH*_, *c*_*OT-OT*_, and *c*_*OH-OT*_*), A*_*ES*_, and *B*_*ES*_ are adjustable parameters and their values were given in Hsieh et al*.*^22^. Afterward, the activity coefficient of component *i* in mixture *S* is determined from:

23 $$\ln \gamma_{i} = n_{i} \sum\limits_{t}^{NHB,OH,OT} {\sum\limits_{{\sigma_{n} }} {p_{i}^{t} \left( {\sigma_{n}^{t} } \right)\left( {\ln \Gamma_{S}^{t} \left( {\sigma_{n}^{t} } \right) - \ln \Gamma_{i}^{t} \left( {\sigma_{n}^{t} } \right)} \right)} } .$$

### Flory–Huggins theory

In this study, a semi-predicative version of the Flory–Huggins model was incorporated based on the Hansen solubility parameters. In Flory–Huggins theory, activity coefficient of component *i* in mixture is obtained from^25^:

24 $$\ln \gamma_{i} = \ln \frac{{\phi_{i} }}{{x_{i} }} + 1 - \frac{{\phi_{i} }}{{x_{i} }} + 2V_{i} \sum\limits_{j} {\chi_{ij} \phi_{j}^{2} } - V_{i} \sum\limits_{j} {\sum\limits_{k} {\phi_{j} \phi_{k} \chi_{jk} } }$$

In the above equation, $\phi$ is the volume fraction ($\phi_{i} = \frac{{x_{i} V_{i} }}{{\sum\nolimits_{i} {x_{i} V_{i} } }}$) and V is the molar volume.$\chi$ is the Flory–Huggins interaction parameter obtained from the Hansen solubility ($\delta$) contributions in the forms non-polar (dispersion) forces (*d*), polar forces (*p*) and hydrogen-bonding (*h*) effects as follows^27^:

25 $$\chi_{ij} = \frac{{V_{i} }}{RT}\left( {\left( {\delta_{d,i} - \delta_{d,j} } \right)^{2} + 0.25\left( {\delta_{p,i} - \delta_{p,j} } \right)^{2} + 0.25\left( {\delta_{h,i} - \delta_{h,j} } \right)^{2} } \right)$$

The Hansen solubility parameters and their contributions were obtained by group contribution methods according to the following equations^28^:

26 $$\delta_{d} = \frac{{\sum\nolimits_{i} {F_{d,i} } }}{{\sum\nolimits_{i} {V_{i} } }}; \, \delta_{p} = \frac{{\left( {\sum\nolimits_{i} {F_{p,i}^{2} } } \right)^{0.5} }}{{\sum\nolimits_{i} {V_{i} } }}; \, \delta_{h} = \frac{{\left( {\sum\nolimits_{i} {E_{h,i} } } \right)^{0.5} }}{{\sum\nolimits_{i} {V_{i} } }}; \, \delta_{t}^{2} = \, \delta_{d}^{2} + \, \delta_{p}^{2} + \, \delta_{h}^{2}$$

The $F_{d,i}$, $F_{p,i}$ ,$E_{h,i}$ and $V_{i}$ values were extracted from Barton^28^.

### Solid–liquid equilibria

In solid–liquid equilibria, the solid solubility in liquid phase is calculated according to the following expression:

27 $$\ln x_{i} = \frac{{\Delta H_{m} }}{R}\left( {\frac{1}{{T_{m} }} - \frac{1}{T}} \right) - \frac{{\Delta C_{P} }}{R}\left( {1 - \frac{{T_{m} }}{T} - \ln \frac{T}{{T_{m} }}} \right) - \ln \gamma_{i}$$

where $x_{i}$ and $\gamma_{i}$ stand the solubility and activity coefficient of compound *i*. The activity coefficient in the above expression was computed from the considered models as described before. $\Delta H_{m}$, $\Delta C_{P}$ and $T_{m}$ represent the fusion enthalpy, the heat capacity of phase change between solid and liquid phases and the melting point temperature, respectively. In the current study, the second term of Eq. (27) was neglected ($\Delta C_{P} = 0$).

### Partition coefficient

When the equilibrium condition between two immiscible liquid phases establishes, the components distribute between two phases. The distribution of component *i* between two phases *α* and *β* measured by partition coefficient as follows^15^:

28 $$K_{i}^{\alpha ,\beta } = \frac{{x_{i}^{\alpha } }}{{x_{i}^{\beta } }} = \frac{{\gamma_{i}^{\beta } }}{{\gamma_{i}^{\alpha } }}$$

where $x_{i}^{\alpha }$ and $x_{i}^{\beta }$ are mole fractions of component *i* in phases α and β; and their activity coefficients, $\gamma_{i}^{\alpha }$ and $\gamma_{i}^{\beta }$, respectively. Therefore, the octanol/water partition coefficient for component *i* ($K_{OW,i}$) calculates from^15^:

29 $$\log K_{OW,i} = \log \left( {\frac{{C_{o,W} \gamma_{i}^{W,\infty } }}{{C_{o,O} \gamma_{i}^{O,\infty } }}} \right)$$

where $C_{o,O}$ and $C_{o,W}$ are total concentrations in octanol-rich and water-rich phases. The $\gamma_{i}^{O,\infty }$ and $\gamma_{i}^{W,\infty }$ are activity coefficients of component *i* in octanol-rich and water-rich phases at dilute concentration. The default values for $\frac{{C_{o,W} }}{{C_{o,O} }}$ is 0.151. The octanol-rich phase is composed from 27.5 mol% water and 72.5 mol% octanol. The water-rich phase is free of octanol.

### Cocrystal formation

The three-phases diagram for a drug and an API with cocrystal (CC) formation includes three lines named solubility lines, API/solvent and drug/solvent, and cocrystal line. The solubility lines of drug and API in solvent are determined from solubility calculations of drug/API in mixture according to Eq. (27) in corporation with the considered models. The cocrystal formation is identified by a chemical reaction between the drug (A) and the API (B) as follows^29,30^:

30 $$aA + bB\overset {K_{CC} } \longleftrightarrow A_{a} B_{b}$$

where *a* and *b* are stoichiometric coefficient of substances A and B in the cocrystal. In the above equations, the *K*_*cc*_ is solubility product and are computed by the following equation:

31 $$K_{CC} = (x_{A} \gamma_{A} )^{a} \times (x_{B} \gamma_{B} )^{b}$$

The activity coefficients in Eq. (31) computed from the examined model. The solubility product (*K*_*CC*_) is depend only on temperature and independent to solvent type. By knowing solubility product at single point, it can be applied to other conditions. After obtaining solubility product for desired system, the invariant points as intersections of cocrystal line and solubility line were computed by simultaneous solvation of Eqs. (27) and (31). Afterward, the cocrystal region is determined by varying drug mole fraction between two invariant points and obtaining API mole fraction from Eq. (31).

### Statistical analysis

In order to explore model precision in comparison to experimental data, several statistics were applied such as absolute average percentage deviation (% AAD), root mean square error (RMSE), mean square error (MSE), normalized root mean square error (NRMSE) and normalized mean square error (NMSE). MSE, NRMSE and NMSE were obtained from goodness of Fit function in MATLAB programming software. Absolute average percentage deviation was calculated as following equations:

33 $$\% AAD = \frac{1}{n}\sum\limits_{i} {\left| {\frac{{\Omega_{i,cal} - \Omega_{i,\exp } }}{{\Omega_{i,\exp } }}} \right|} \times 100$$

where $\Omega_{cal}$ are $\Omega_{\exp }$ calculated and experimental data of desired properties and *n* is number of experimental data. The root mean square error (RMSE) was obtained as follows:

34 $$RMSE = \sqrt {\left| {\frac{{\sum\nolimits_{i} {\left( {\Omega_{i,cal} - \Omega_{i,\exp } } \right)^{2} } }}{n}} \right|} .$$

## Results and discussion

The object of this section is to evaluate the performances of the COSMO-SAC (2002), the COSMO-SAC (2010) and the Flory–Huggins models for pharmaceutical compounds, which mostly are complicated/massive molecules containing electronegative atoms such as N, O, and S; and complicated bonds between atoms such as hydrogen bonding. The considered properties are solubilities of pharmaceutical compounds in pure solvent and solvent mixtures. The octanol/water partition coefficient and cocrystal formation of pharmaceutical compounds are other examined properties. In order to conduct the study, firstly, the COSMO files from DMol^3^ were required. Thus, the COSMO files prepared for 15 solvents and 35 pharmaceutical compounds from DMol^3^ modules in Materials Studio 2017 software. In performing the COSMO file, density function was chosen to GGA (VWN-BP) by quality fine. In electronic options, multipolar expansion was selected octupole. The calculations run at four parallel cores. Other options set to default values in DMol^3^.

**Figure 1:**
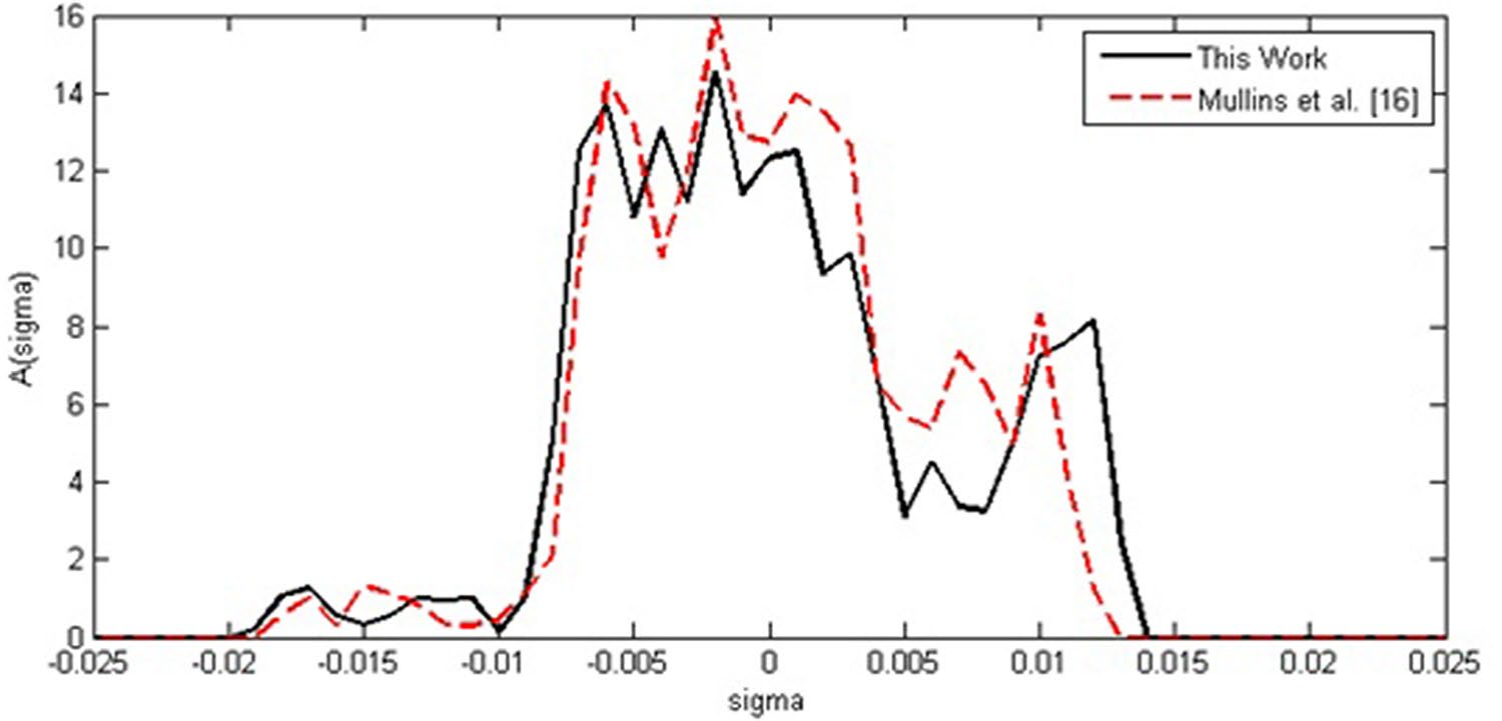
Generated sigma profiles for acetyl salicylic acid in comparison to Mullins et al*.*^16^.

**Figure 2:**
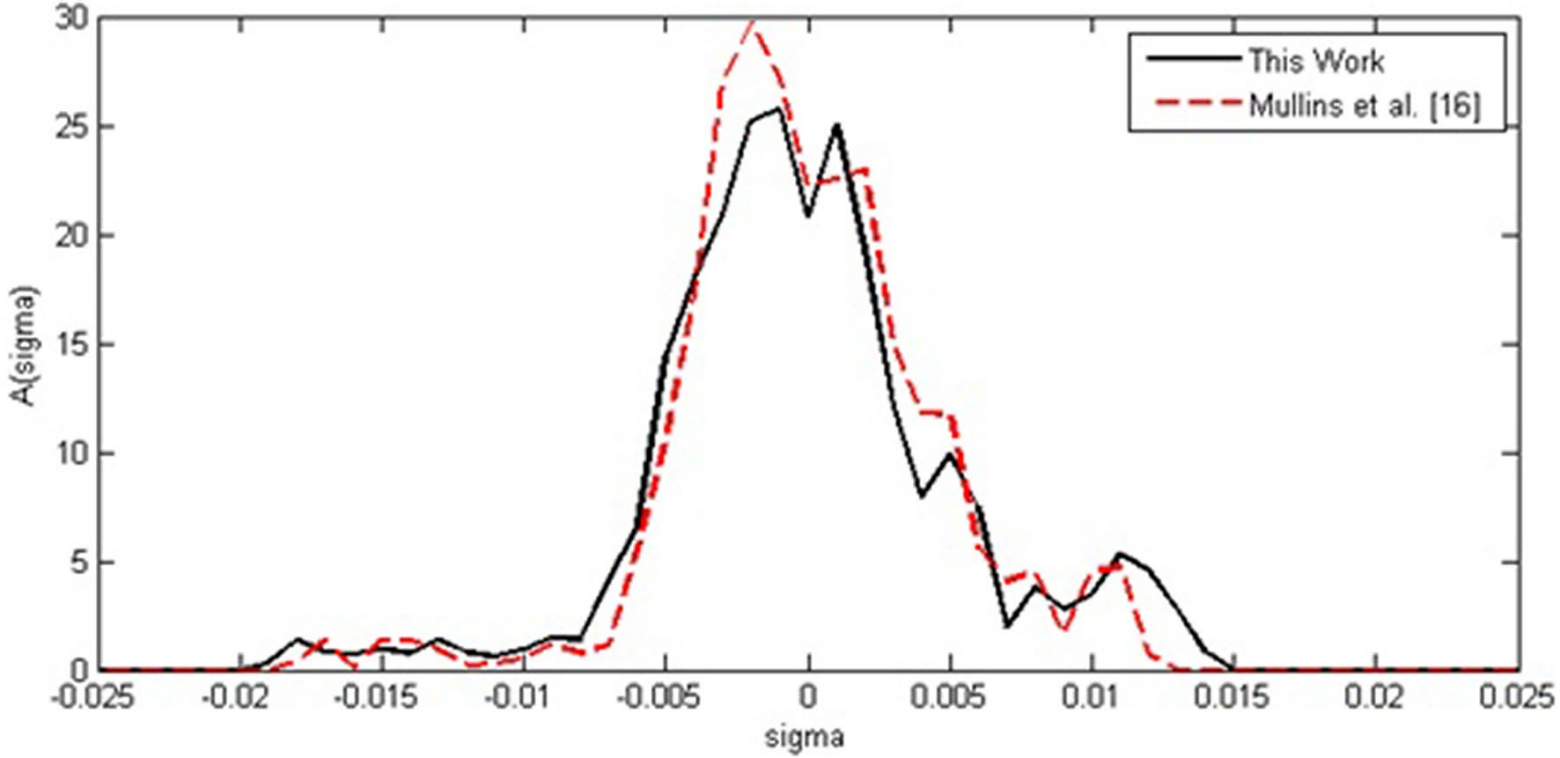
Generated sigma profiles for Ibuprofen in comparison to Mullins et al*.*^16^.

After generating the COSMO file, it is time to test sigma profiles obtained in the current study by reported sigma profiles by other studies. Figures 1 and 2 compare sigma profiles generated in current studies for ibuprofen and acetyl salicylic acid in comparison to sigma profiles in the database provided by Mullins et al*.*^16^. Based on Figs. 1 and 2, the same trends between results in this study and Mullins et al*.*^16^ were observed. The small departures between two curves originated from the software version and the sigma profile generation program.

After generating the sigma profiles and providing the COSMO-SAC computation program for the activity coefficient, the solubilities in the binary and ternary systems were calculated and compared by experimental data obtained from the literature.

**Figure 3:**
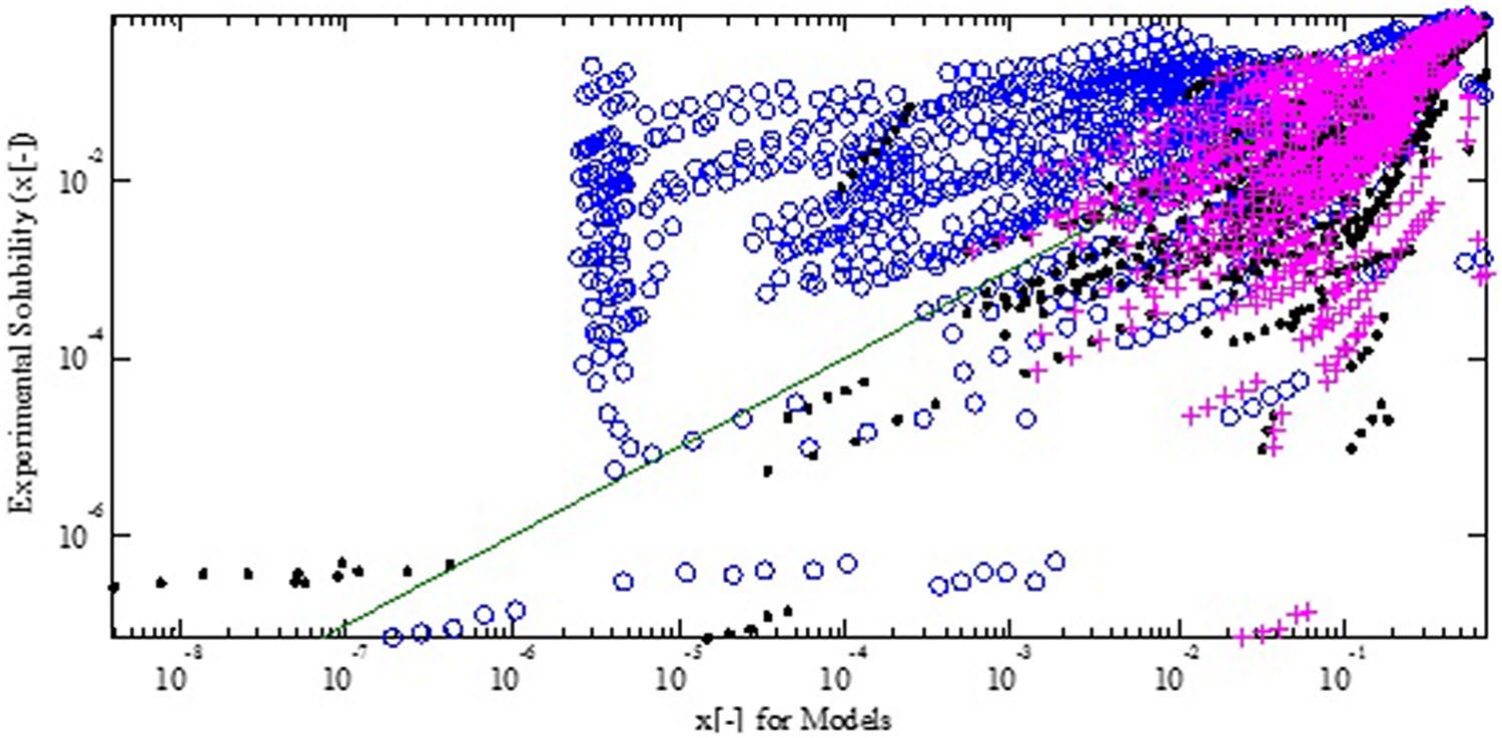
Parity plot of solubility in pure solvent (mole fraction) from the COSMO-SAC (2002) (dot symbol), the COSMO-SAC (2010) (plus symbol) and Flory–Huggins model (circle symbol) in comparison to experimental data.

Figure 3 shows the parity plots of experimental solubility in the pure solvents in comparison to calculated solubilities from the COSMO-SAC (2002), the COSMO-SAC (2010), and the Flory–Huggins models. The mean square error (MSE), normalized root mean square error (NRMSE), and normalized mean square error (NMSE) for the COSMO-SAC (2002) model are 0.0136, 0.0349, and 0.0685. The MSE, NMSE, and NRMSE for the COSMO-SAC (2010) are 0.0187, − 0.2718, and − 0.1277. While MSE, NMSE, and NRMSE for the Flory–Huggins model are 0.0360, − 1.2337, and − 0.4946. According to Fig. 3, it is observed that the Flory–Huggins model under predicts the solubility data. The examined pharmaceutical compounds contain a wide variety of components made of small to long-chain molecules. The pharmaceutical compounds compose of atoms C, H, N, O, S, F, and Cl, which joint by covalent bonds and stronger bonds such as hydrogen bonding. The reported statistics imply on the relatively acceptable performance of the COSMO-SAC (2002) regarding to the COSMO-SAC (2010). The comparison between accuracy of COSMO-SAC (2002) and COSMO-SAC (2010) seems to be inconsistent with those reported in the literature^15^. The accuracy of these two COSMO-SAC models has been comprehensively examined through a very large dataset, containing 29,173 data points of infinite dilution activity coefficient and 139,921 VLE data points of 6940 binary mixtures^31^. The mentioned inconsistency arises from different universal constants implemented in sigma profile generation. The differences in investigated systems attribute the second reason for the observed inconsistency.

It is interesting that the COSMO-SAC (2002) was obtained by only eight universal constant parameters without any further modifications. A list of considered pharmaceutical compounds and their physical properties and references for experimental data were presented in supplementary materials (Table S1).

The Hansen solubility parameters, molar volumes for the Flory–Huggins model and the COSMO molar volume of the examined pharmaceutical compounds and solvents were presented on Table 1. Based on Table1, the molar volume obtained from group contribution method in Barton^28^ and the COSMO calculations have some difference.

**Table 1:** Hansen solubility parameters and molar volumes from group contribution method in comparison to molar volumes obtained from the COSMO calculations.

Substance	$\delta_{t} ({\text{MPa}}^{0.5} )$	$\delta_{d} ({\text{MPa}}^{0.5} )$	$\delta_{p} ({\text{MPa}}^{0.5} )$	$\delta_{h} ({\text{MPa}}^{0.5} )$	V(cm^3^/mol) Flory–Huggins	V(cm^3^/mol) COSMO
1-Propanol	24.5	16	6.8	17.4	75.2	52.64
2-Propanol	23.5	15.8	6.1	16.4	76.8	52.64
Acetic Acid	21.4	14.5	8	13.5	57.1	43.38
Acetone	20	15.5	10.4	7	74.0	49.92
Acetonitrile	24.4	15.3	18	6.1	52.6	38.41
Ethanol	26.5	15.8	8.8	19.4	58.5	40.33
Ethyl Acetate	18.1	15.8	5.3	7.2	98.5	68.14
Heptane	15.3	15.3	0	0	147.4	94.24
Hexane	14.9	14.9	0	0	131.6	82.11
Methanol	29.6	15.1	12.3	22.3	40.7	29.09
Methyl Acetate	18.7	15.5	7.2	7.6	79.7	56.13
Octanol	21	17	3.3	11.9	157.7	112.43
Water	47.8	15.6	16	42.3	18.0	15.24
2-Phenylacetamide	27.89	22.54	16.38	1.2	53.63	98.25
4-Methylphthalic anhydride	32.45	27.18	17.7	1.02	66.0	103.50
Aceclofenac	28.02	26.64	8.65	0.79	121.83	213.03
Acetaminophen	28.24	23.37	15.75	1.85	60.07	104.32
Acetylsalicylic acid	29.06	27.45	9.46	1.15	70.63	116.83
Atenolol	21.38	20.23	6.88	0.81	161.3	190.11
Atropine	27.78	26.84	7.11	1.03	103.07	195.58
Benzamide	35.25	25.7	24.05	1.76	36.53	86.76
Camphor	20.59	19.29	7.21	0.29	106.7	111.53
Capecitabine	24.65	22.83	9.26	0.83	205.94	225.47
Cefixime	30.18	27.82	11.67	0.86	190.56	264.81
Cephalexin	33.22	29.67	14.91	0.99	111.46	219.59
Cimetidine	27.39	22.85	15.1	0.61	162.3	176.03
Deferiprone	22.59	19.64	11.09	1.25	83.97	95.13
Flurbiprofen	24.56	23.03	8.49	0.86	82.33	164.03
Hydroquinone	43.64	31.25	29.89	5.87	23.84	75.95
Isoniazid	44.85	27.74	35.19	1.89	45.53	93.62
Lamotrigine	40.17	29.09	27.67	1.23	90.26	147.47
Meclofenamic acid	25.08	23.52	8.69	0.78	106.43	177.91
Pentoxifylline	23.76	21.49	10.14	0.4	187.2	184.38
Pindolol	23.46	21.41	9.55	0.92	129.37	177.38
Pnitrobenzamide	36.71	26.8	25.05	1.28	55.33	104.25
Vinpocetine	32.28	31.84	5.28	0.43	108.8	235.71
Benzocaine	27.88	26.5	8.64	0.91	77.33	115.91
Borneol	19.15	18.54	4.68	0.93	106.67	115.13
Carvedilol	25.27	24.45	6.32	0.9	146.27	274.61
Ibuprofen	18.35	18.09	3.07	0.5	140.43	154.87
Isoborneol	20.06	19.48	4.72	0.94	105.67	114.97
Salicylic acid	30.52	25.83	16	2.94	41.2	90.21

Table 2 reports the COSMO-SAC (2002), the COSMO-SAC (2010), and the Flory–Huggins results for some pharmaceutical compounds categorized by the solvent type and sorted according to absolute average deviations (AAD%). The RMSE results for the COSMO-SAC (2002), the COSMO-SAC (2010), and the Flory–Huggins models were also reported in Table 2. Based on Table 2, the predictive model of the COSMO-SAC (2002) has a wide range of errors that are in agreement with errors reported by Hsieh et al*.*^15^. The COSMO-SAC (2010) and the Flory–Huggins have larger errors compared to the COSMO-SAC (2002).

**Table 2:** The results of solubility from the COSMO-SAC model (2002) for some considered pharmaceutical compounds in comparison to Flory–Huggins model and the COSMO-SAC (2010).

Drug	Solvent	RMSE COSMO-SAC (2002) model	RMSE Flory–Huggins model	RMSE COSMO-SAC (2010) model	AAD% COSMO-SAC model
4-Methylphthalic anhydride	Methyl acetate	0.0017	0.3106	0.0215	0.47
Atropine	Ethanol	0.0072	0.0769	0.0252	7.25
Acetyl salicylic acid	Ethanol	0.0050	0.1254	0.0324	8.13
Camphor	Ethanol	0.0832	0.0675	0.0625	10.40
Isoborneol	Acetone	0.0529	0.2311	0.1252	12.02
Vinpocetine	Ethyl acetate	0.0021	0.0108	0.0027	12.16
Salicylic acid	Methanol	0.0285	0.1375	0.0796	20.21
Acetyl salicylic acid	Octanol	0.011	0.0815	0.0400	21.35
Atenolol	Octanol	0.0011	0.0004	0.0046	21.40
4-Methylphthalic anhydride	Acetonitrile	0.0475	0.2115	0.0310	23.92
Atropine	Octanol	0.0245	0.0916	0.0482	25.5
Salicylic acid	Acetic Acid	0.0156	0.0643	0.0271	26.26
Camphor	Acetone	0.1655	0.2131	0.1633	28.05
Isoborneol	Ethanol	0.1210	0.1359	0.1037	28.81
Ibuprofen	Octanol	0.1145	0.0349	0.0332	30.52
Dapsone	Methyl Acetate	0.0126	–	0.0162	30.82
4-Methylphthalic anhydride	Acetone	0.0732	0.2585	0.0411	34.39
Pindolol	Octanol	0.0011	0.0023	0.0001	46.76
Flurbiprofen	Octanol	0.0769	0.113	0.0388	51.91
Acetaminophen	Ethanol	0.0352	0.0563	0.0112	51.96
Pindolol	Hexane	2.50E−07	1.00E−04	0.0000	63.05
Ibuprofen	Ethanol	0.1918	0.0741	0.1246	67.76
Aceclofenac	Acetone	0.0549	0.0812	0.0609	71.11
Aceclofenac	Methanol	0.019	0.0437	0.0105	72.28
Lamotrigine	Acetonitrile	0.002	0.0018	–	78.78
Atenolol	Hexane	3.28E−07	0.0011	0.0000	91.5
Acetyl salicylic acid	2-Propanol	0.0423	0.0536	0.0189	93.87
Pentoxifylline	Octanol	0.1132	0.1246	0.1193	96.2
Benzamide	Methanol	0.1024	0.0969	0.1393	97.43
Meclofenamic acid	Water	0.0361	0.2142	0.1632	99.33
*p*-Nitrobenzamide	Water	0.0013	0.0007	0.0478	99.43
Borneol	Acetone	0.1248	0.0922	0.1250	101.77
Sulfamethazine	Water	4.86E−05	–	0.1250	116.51
Probenecid	Acetone	0.0298	–	0.0192	116.99
Dapsone	Methanol	0.0165	0.0118	0.0979	119.69
Flurbiprofen	Ethanol	0.1533	0.1038	0.0789	124.04
Acetaminophen	Octanol	0.0528	0.0091	0.0205	135.9
Meclofenamic acid	Ethanol	0.1813	0.1428	0.0608	142.16
Benzamide	Acetonitrile	0.0631	0.0037	0.1367	145.76
Acetaminophen	2-Propanol	0.1029	0.0606	0.0160	157.2

According to Table 2, pharmaceutical compounds containing H, C and O with the lowest hydrogen bonding numbers have the lower error. Besides, the structure of molecule has a remarkable influence on accuracy. In the case of acetaminophen and acetyl salicylic acid, by solvent replacement from ethanol to acetone, deterioration in model prediction was observed. The impact of eliminating F atom from flurbiprofen observes in the lower error reported for ibuprofen. Although borneol and isoborneol have the same chemical formula, the accuracy of the COSMO-SAC (2002) for them is entirely different. The above studies implied that molecular structure, atoms, and intermolecular interaction must be widely incorporated into the COSMO-SAC model. Since, the COSMO-SAC (2002) provides better approximations of solubility in examined systems, we prefer utilizing the original COSMO-SAC (2002) in our further investigation on the binary and ternary systems. Afterward, two models, the COSMO-SAC (2002) and the Flory–Huggins models were considered for the octanol/water partition coefficient and cocrystal formation.

**Figure 4:**
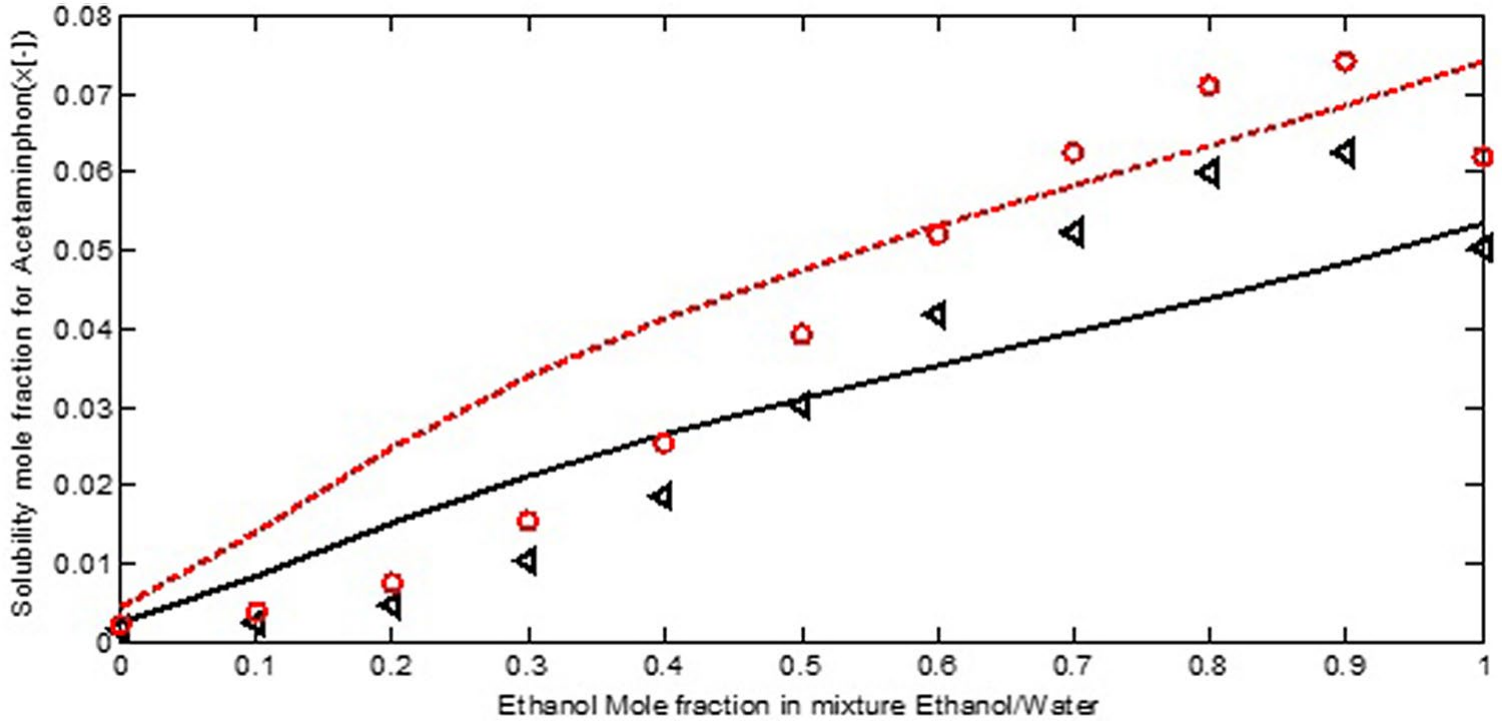
The experimental (symbol) and calculated (line) solubility of acetaminophen in ethanol/water mixtures at 293.15 K(triangular symbol) and 303.15 K (circle symbol)^32^.

Afterward, the ternary systems of pharmaceutical compounds in binary solvents were also examined. On the basis of Table 2, two pharmaceutical compounds, acetaminophen and salicylic acid, were suggested. Acetaminophen consists of 20 atoms H, C, N, and O and two functional groups, OH and NH. Salicylic acid consists of 16 atoms H, C, and O, and two functional groups, OH and COOH. Figure 4 presents the comparison between the experimental and calculated solubilities of acetaminophen in ethanol/water mixtures as a function of ethanol mole fraction at two temperatures, 293.15 and 303.15 K. According to Fig. 4, a good agreement between experimental data and the COSMO-SAC calculations observe. The observed trends of the COSMO-SAC as a function temperature match with the reported experiments.

**Figure 5:**
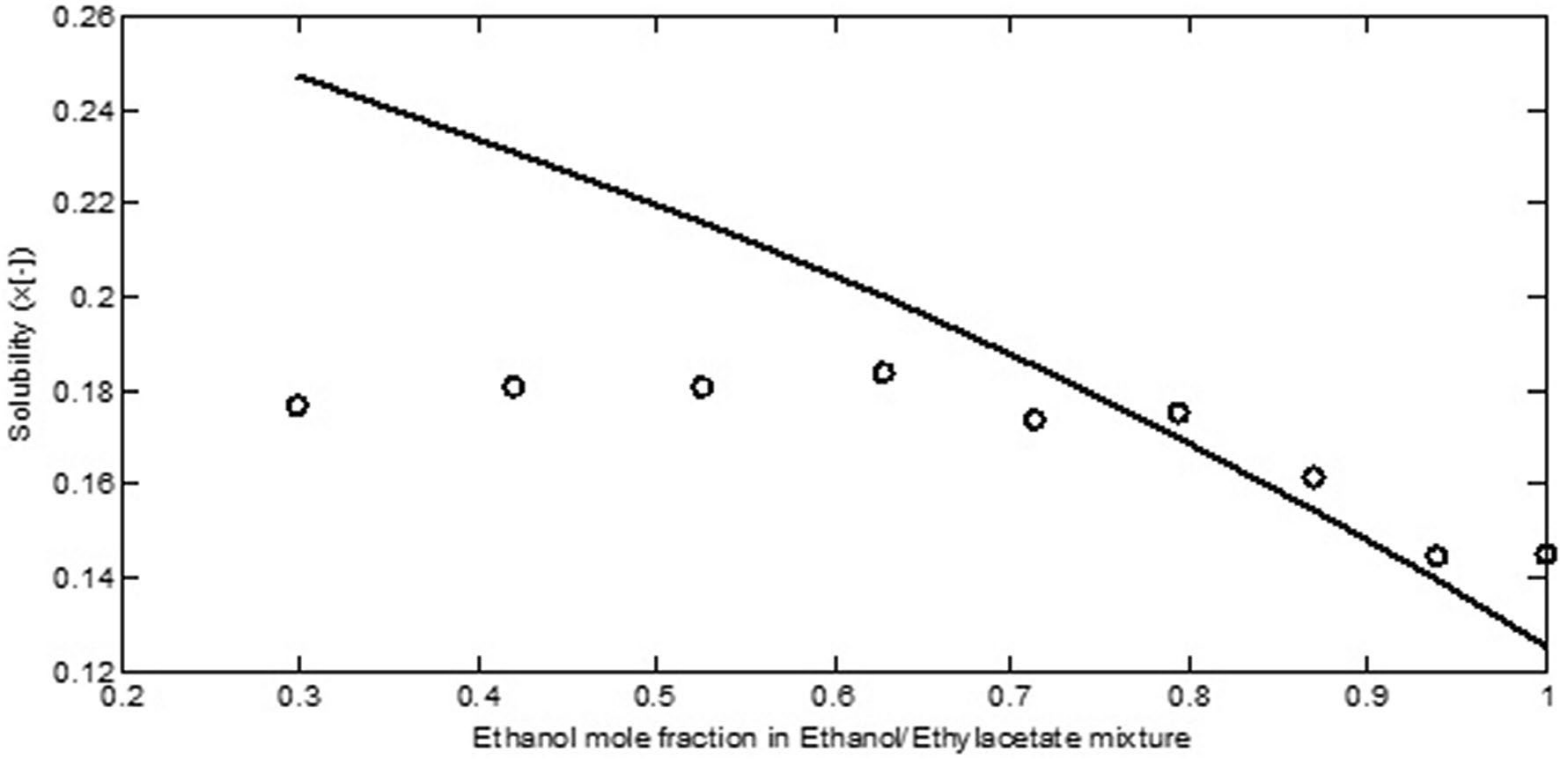
The experimental (symbol) and calculated (line) solubility of salicylic acid in ethanol/ethyl acetate mixture^33^.

Figure 5 shows the calculated solubility of salicylic acid in ethanol/ethyl acetate mixture compared to experimental data. On the basis of Fig. 5, a departure from experimental data was observed at higher ethyl acetate mole fraction. The ethyl acetate has a functional group COO which its interaction with COOH in salicylic acid has been ignored in the COSMO-SAC (2002).

The octanol/water partition coefficients for some pharmaceutical compounds obtained from the COSMO-SAC model. In Table 3, the results of the octanol/water partition coefficient from the COSMO-SAC model compared to experimental data from the national library of medicine^34^. The MSE, NMSE, and NRMSE are 2.36, 0.1416, and 0.0735. The RMSEs for the COSMO-SAC and the Flory–Huggins are 1.25 and 4.45. On the basis of Table 3, the various accuracies obtained regarding activity ratio in the octanol/water partition coefficient. In the octanol/water partition coefficient, if the errors in the numerator and denominator cancel each other out, a good accuracy between the COSMO-SAC computation and experiment is harvested. Otherwise, the discrepancies in obtained errors were seen. It is possible that the COSMO-SAC model fails for solubility prediction (such as dapsone) but presents a reasonable estimation of the octanol/water partition coefficient due to the above discussions. As observed from Table 3, the simple molecules made of H, C, and O by only hydrogen bonding have better performance in the COSMO-SAC predictions. On the basis of Table 3, the octanol/water partition coefficients obtained from the Flory–Huggins model are farm from experimental data.

**Table 3:** The calculated and experimental octanol/water partition coefficient for some pharmaceutical compounds.

Substance	log K_OW,COSMO-SAC_	log K_OW,Flory–Huggins_	log K_OW,exp_
Aceclofenac	1.57	− 1.31	2.17
Acetaminophen	0.02	−1.56	0.46
Atropine	0.65	− 1.74	1.83
Camphor	1.16	− 1.4	2.38
Cefixime	− 2.22	− 1.04	− 0.40
Celecoxib	2.83	− 2.48	3.53
Dapsone	0.33	− 1.29	0.97
Deferiprone	− 0.94	− 1.14	− 0.77
Flurbiprofen	1.34	− 1.73	4.16
Hydroquinone	0.57	− 3.4	0.59
Isoniazid	− 0.98	− 3.22	− 0.70
Lamotrigine	0.82	− 2.13	2.57
Meclofenamic acid	2.83	− 2.17	5.00
Pindolol	1.43	− 3.70	1.75
*p*-Nitrobenzamide	0.07	− 0.89	0.82
Sulfamethazine	0.99	− 1.69	0.89
Borneol	1.78	− 1.3	3.24
Carvedilol	2.66	− 0.83	4.19
Ibuprofen	2.03	− 1.91	3.97
Isoborneol	2.35	− 3.00	3.24
Sulfacetamide	− 0.04	− 1.68	− 0.96
Trifloxystrobin	3.86	− 3.4	4.50

**Figure 6:**
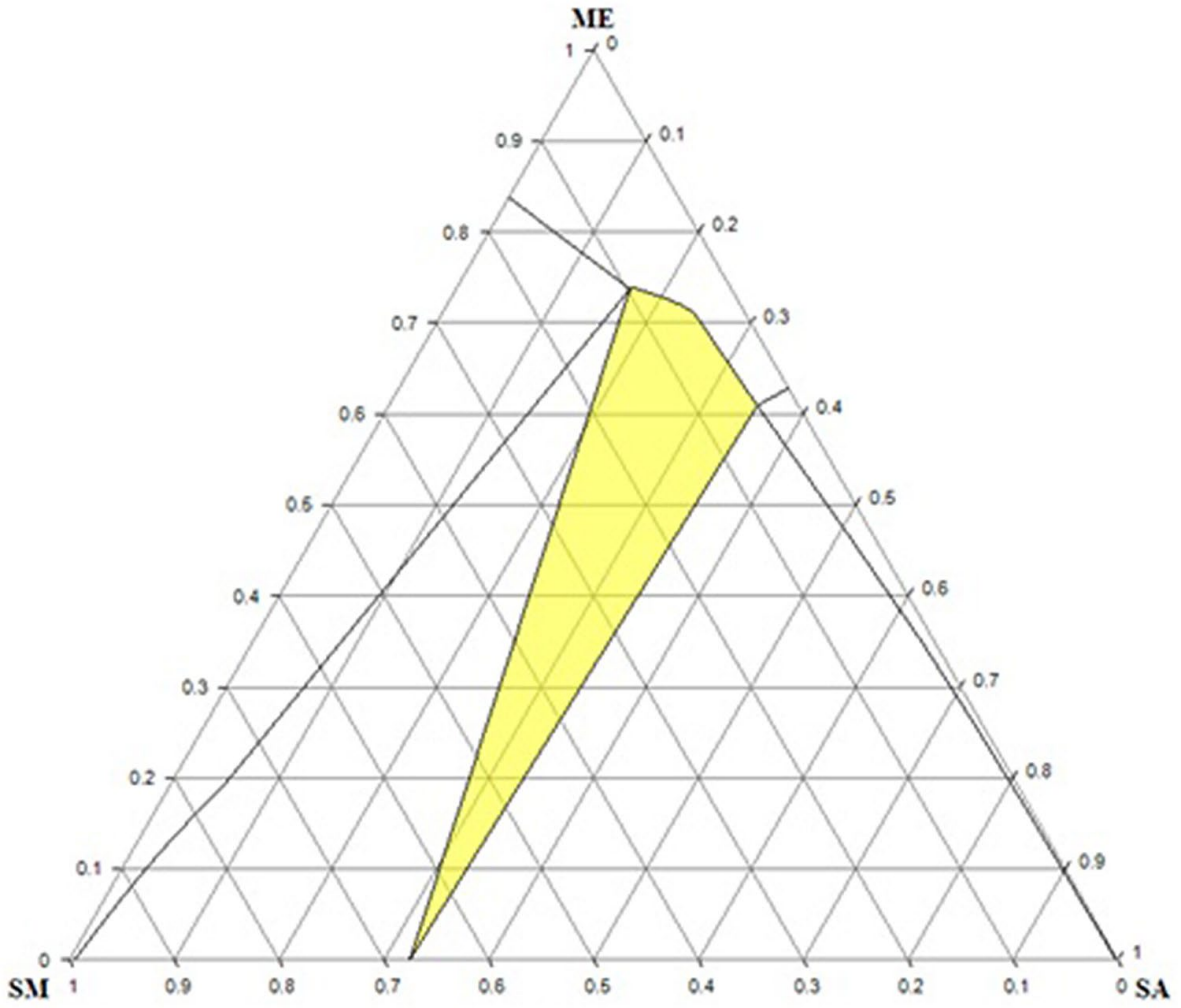
Ternary phase diagram of the system sulfamethazine (SM) /salicylic acid (SA)/methanol (ME) in mass fraction obtained by the COSMO-SAC (2002) model at 283.15 K. The solid lines represent solubility lines and highlighted area shows cocrystal region.

**Figure 7:**
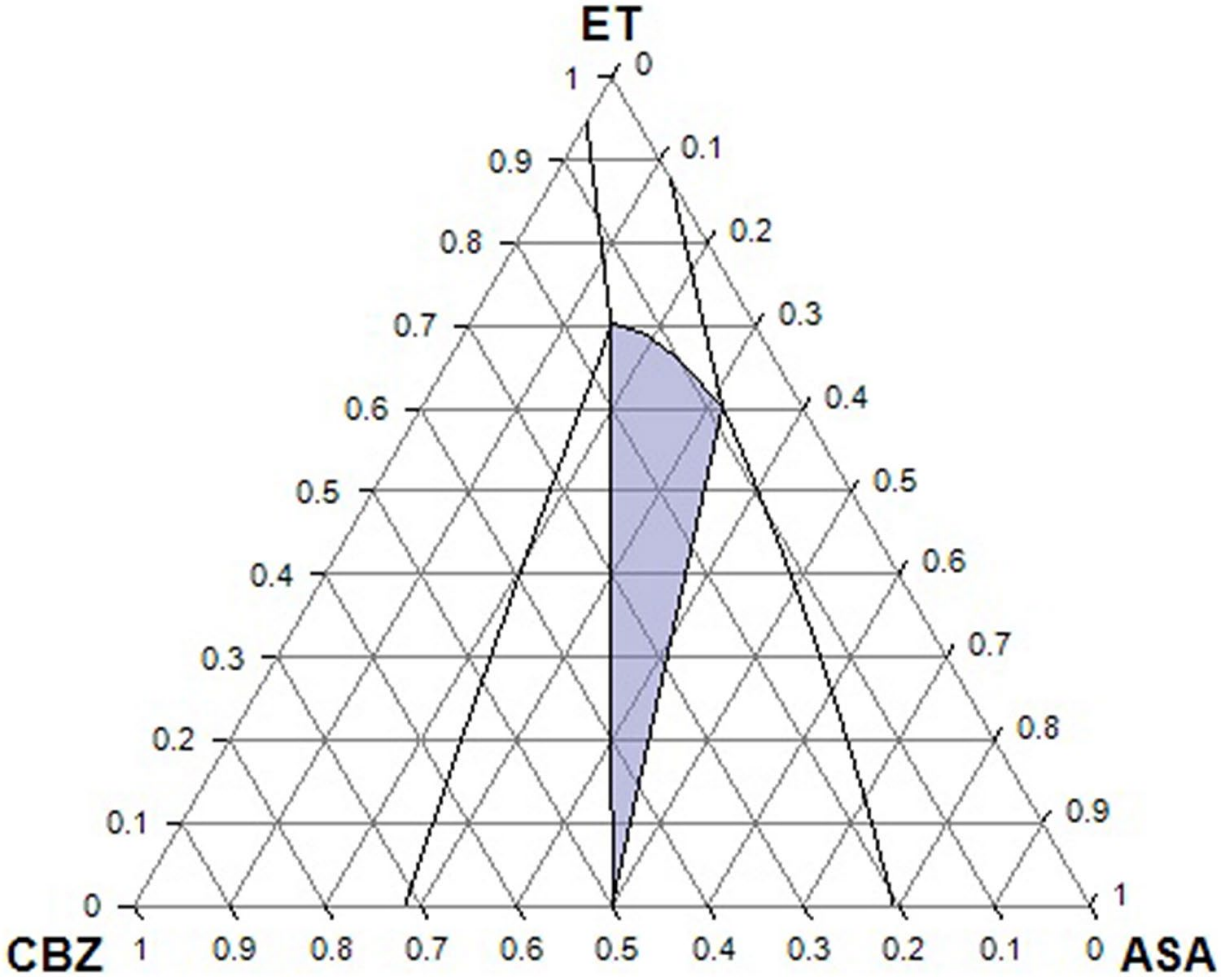
Ternary phase diagram of CBZ/ASA/ET in mole fraction at 298.15 K. Solid lines represent solubility line by the COSMO-SAC (2002). The highlighted region shows cocrystal formation by the COSMO-SAC (2010).

In order to investigate a more complex system, a three-phases diagram of ternary system is explored by considering the sulfamethazine/salicylic acid cocrystal formation in methanol at 283.15 K, which studied by Ahuja et al*.*^35^. Details of calculation and methods were described in “Cocrystal formation” section. After performing the computation by the COSMO-SAC (2002), a triangular diagram of the considered system was plotted by a free software named ProSim Ternary Diagram. On the basis of Fig. 6 and experimental plots in Ahuja et al*.*^35^, some differences between experiments and the COSMO-SAC calculations were observed. The cocrystal region for SM/SA predicted by the COSMO-SAC is wider, while experimental data imply on the narrow region. The solubility line of SM in SA + ME mixture expanded in the COSMO-SAC model in comparison to experiments which interpreted by the COSMO-SAC ability in the considered system. The predicted solubility line of SA in the SM + SA is appropriately closer to the reported experimental data which indicates the good performance of the COSMO-SAC for SA. The reported inconsistencies in observed results originated from molecular structure, constituent atoms, and their interactions. The electronegative atoms S and N in sulfamethazine create the observed discrepancies, while their contributions were not considered in the COSMO-SAC (2002) model. The ternary phase diagram carbamazepine (CBZ)/acetylsalicylic acid (ASA) in ethanol (ET) at 298.15 K were computed by the COSMO-SAC (2002) and plotted in Fig. 7. Veith et al*.*^29^ studied the CBZ/ASA/ET by PC-SAFT EOS. According to Veith et al*.*^29^, the PC-SAFT EOS without binary interaction parameters estimated the narrow cocrystal region and low solubilities. Whilst the COSMO-SAC (2002) predicts higher solubilities and wider cocrystal region. By comparison the COSMO-SAC (2002) calculations to the PC-SAFT EOS by considering binary interaction parameters and experimental data Veith et al*.*^29^, a reasonable agreement observes between the COSMO-SAC (2002) and reported data.

## Conclusions

The COSMO-SAC as a predictive model has been gained a great attention in thermodynamic modeling and phase equilibria considerations. The eight universal parameters and predefined atomic radiuses for C, H, O, S, N, F, and Cl are the general basis of the COSMO-SAC model. In the current study, the COSMO-SAC model implemented in solid–liquid phase equilibria in form of solubility data in binary and ternary systems, octanol/water partition coefficient, and cocrystal studies. For more comparison, the COSMO-SAC model was also compared with the Flory–Huggins model. The obtained results implied that molecular structure, constituent atoms, functional group, and their interactions have remarkable impacts on the obtained results. In general, the simple molecules made of atoms H, C, and O under special condition, atom N by simple covalent and hydrogen bonding interactions can be deliberated by the COSMO-SAC model. The presence of other atoms such as F and S and other functional groups such as COO and COOH made complex systems. This complexity provides some opportunities to modify the original the COSMO-SAC model.

## Supplementary information

Supplementary Information.
