# Supplementary material for: Investigation of COSMO-SAC model for solubility and cocrystal formation of pharmaceutical compounds
Source: Sci Rep. 2020 Nov 16;10:19879. doi: 10.1038/s41598-020-76986-3 (PMC7670437; doi:10.1038/s41598-020-76986-3)
Supplement: Supplementary file 1 — Supplementary Information. [file 41598_2020_76986_MOESM1_ESM.docx]

**Supplementary materials**

**Investigation of COSMO-SAC Model for Solubility and Cocrystal Formation of Pharmaceutical Compounds**

Samane Zarei Mahmoudabadi, Gholamreza Pazuki^*^

^*^ Corresponding author. Tel.: + 98 21 64543159; fax: + 98 21 66405847; Email: ghpazuki@aut.ac.ir

Department of Chemical Engineering, Amirkabir University of Technology (Tehran Polytechnic), Tehran, Iran

Table S1. The melting point and enthalpy of fusion for examined pharmaceutical compounds.

| Number | Name | T_m_ [K] | $\Delta H_{m}$[J/mol]  | Reference |
| --- | --- | --- | --- | --- |
| 1 | 2-Phenylacetamide | 406.67 | 2.53E+04 | [1] |
| 2 | 4-Methylphthalic Anhydride | 361.47 | 1.93E+04 | [2] |
| 3 | Aceclofenac | 424.55 | 5.20E+04 | [3] |
| 4 | Acetaminophen | 442.35 | 2.75E+04 | [4] |
| 5 | Acetylsalicylic Acid | 414.15 | 3.55E+04 | [4] |
| 6 | Atenolol | 426.10 | 3.87E+04 | [5] |
| 7 | Atropine | 388.50 | 3.55E+04 | [6] |
| 8 | Benzamide | 401.10 | 1.92E+04 | [7] |
| 9 | Borneol | 478.30 | 8.29E+03 | [8] |
| 10 | Isoborneol | 482.70 | 8.80E+03 | [8] |
| 11 | Camphor | 451.50 | 6.82E+03 | [8] |
| 12 | Capecitabine | 393.05 | 2.72E+04 | [9] |
| 13 | Carvedilol | 398.68 | 4.84E+04 | [10] |
| 14 | Cefixime Trihydrate | 500.15 | 2.39E+04 | [11] |
| 15 | Celecoxib | 431.15 | 37908.178 | [12, 13] |
| 16 | Cephalexin Monohydrate | 500.15 | 2.39E+04 | [11] |
| 17 | Cimetidine | 414.15 | 3.55E+04 | [4] |
| 18 | Dapsone | 454.41 | 2.86E+04 | [14] |
| 19 | Deferiprone | 545.15 | 3.30E+04 | [11] |
| 21 | ﬂurbiprofen | 386.20 | 2.68E+04 | [6] |
| 24 | Hydroquinone | 445.00 | 2.71E+04 | [15] |
| 25 | Ibuprofen | 347.70 | 2.77E+04 | [6] |
| 26 | Isoniazid | 445.84 | 2.79E+04 | [16] |
| 27 | Lamotrigine | 491.00 | 3.90E+04 | [11] |
| 30 | Meclofenamic Acid | 402.50 | 1.83E+04 | [6] |
| 32 | Pentoxifylline | 376.80 | 3.66E+04 | [6] |
| 33 | P-nitrobenzamide | 473.45 | 3.30E+04 | [17] |
| 34 | Probenecid | 472.05 | 4.09E+04 | [18] |
| 37 | Pindolol | 423.60 | 6.06E+04 | [5] |
| 41 | Salicylic Acid | 432.65 | 2.71E+04 | [19] |
| 42 | Sulfacetamide | 455.20 | 2.98E+04 | [20] |
| 43 | Trifloxystrobin | 345.67 | 3.17E+04 | [21] |
| 44 | Vinpocetine | 422.15 | 3.74E+04 | [22] |
| 45 | Sulfamethazine | 469.20 | 3.77E+04 | [23, 24] |
| 46 | Benzocaine | 363.40 | 2.10E+04 | [10] |

**Reference**

1. Li, Y., K. Wu, and L. Liang, *Solubility behavior of 2-phenylacetamide in sixteen pure solvents and dissolution properties of solution.* Journal of Molecular Liquids, 2019. **291**. 10.1016/j.molliq.2019.111264.

2. Yu, Y., et al., *Experiment, correlation and molecular simulation for solubility of 4-methylphthalic anhydride in different organic solvents from T = (278.15 to 318.15) K.* Journal of Molecular Liquids, 2019. **275**: p. 768-777. 10.1016/j.molliq.2018.10.158.

3. Liu, J.Q., et al., *Experimental measurements and modeling of the solubility of aceclofenac in six pure solvents from (293.35 to 338.25) K.* Journal of Chemical and Engineering Data, 2014. **59**(5): p. 1588-1592. 10.1021/je500038u.

4. Matsuda, H., et al., *Determination and prediction of solubilities of active pharmaceutical ingredients in selected organic solvents.* Fluid Phase Equilibria, 2015. **406**: p. 116-123. 10.1016/j.fluid.2015.07.032.

5. Perlovich, G.L., T.V. Volkova, and A. Bauer-Brandl, *Thermodynamic study of sublimation, solubility, solvation, and distribution processes of atenolol and pindolol.* Molecular Pharmaceutics, 2007. **4**(6): p. 929-935. 10.1021/mp070039b.

6. Domañska, U., et al., *pKa and solubility of drugs in water, ethanol, and 1-octanol.* Journal of Physical Chemistry B, 2009. **113**(26): p. 8941-8947. 10.1021/jp900468w.

7. Ouyang, J., et al., *Solubility determination and modelling of benzamide in organic solvents at temperatures from 283.15 K and 323.15 K, and ternary phase diagrams of benzamide-benzoic acid cocrystals in ethanol at 298.15 K.* Journal of Molecular Liquids, 2019. **286**. 10.1016/j.molliq.2019.110885.

8. Chen, J., et al., *Determination and Correlation of Solubility of Borneol, Camphor, and Isoborneol in Different Solvents.* Journal of Chemical and Engineering Data, 2019. **64**(4): p. 1826-1833. 10.1021/acs.jced.9b00045.

9. Zhao, R., et al., *Solubility and dissolution characteristics of capecitabine in pure lower alcohols and water with methanol mixture solvents at atmospheric pressure and different temperatures.* Fluid Phase Equilibria, 2018. **460**: p. 23-35. 10.1016/j.fluid.2017.12.024.

10. Ha, E.S., et al., *Equilibrium solubility and solute-solvent interactions of carvedilol (Form I) in twelve mono solvents and its application for supercritical antisolvent precipitation.* Journal of Molecular Liquids, 2019. **294**. 10.1016/j.molliq.2019.111622.

11. Yousefi Seyf, J. and A. Haghtalab, *Measurement and thermodynamic modeling of the solubility of lamotrigine, deferiprone, cefixime trihydrate, and cephalexin monohydrate in different pure solvents from 283.1 to 323.1 K.* Journal of Chemical and Engineering Data, 2016. **61**(6): p. 2170-2178. 10.1021/acs.jced.6b00163.

12. Jouyban, A., S. Nozohouri, and F. Martinez, *Solubility of celecoxib in {2-propanol (1) + water (2)} mixtures at various temperatures: Experimental data and thermodynamic analysis.* Journal of Molecular Liquids, 2018. **254**: p. 1-7. 10.1016/j.molliq.2018.01.033.

13. Nozohouri, S., et al., *Solubility of celecoxib in N-methyl-2-pyrrolidone + 2-propanol mixtures at various temperatures.* Journal of Molecular Liquids, 2017. **241**: p. 1032-1037. 10.1016/j.molliq.2017.06.080.

14. Li, W., et al., *Solubility measurement, correlation and mixing thermodynamics properties of dapsone in twelve mono solvents.* Journal of Molecular Liquids, 2019. **280**: p. 175-181. 10.1016/j.molliq.2019.02.023.

15. Li, X., et al., *Solubility of hydroquinone in different solvents from 276.65 K to 345.10 K.* Journal of Chemical and Engineering Data, 2006. **51**(1): p. 127-129. 10.1021/je0502748.

16. Heryanto, R., M. Hasan, and E.C. Abdullah, *Solubility of isoniazid in various organic solvents from (301 to 313) K.* Journal of Chemical and Engineering Data, 2008. **53**(8): p. 1962-1964. 10.1021/je800156m.

17. Yuan, Y., et al., *Solubility Determination and Modeling of p-Nitrobenzamide Dissolved in Twelve Neat Solvents from 283.15 to 328.15 K.* Journal of Chemical and Engineering Data, 2019. **64**(4): p. 1840-1850. 10.1021/acs.jced.9b00065.

18. Shi, J., et al., *Solubility Measurement and Correlation of Probenecid in 12 Pure Organic Solvents and Thermodynamic Properties of Mixing of Solutions.* Journal of Chemical and Engineering Data, 2019. **64**(2): p. 624-631. 10.1021/acs.jced.8b00863.

19. Nordström, F.L. and Å.C. Rasmuson, *Solubility and melting properties of salicylic acid.* Journal of Chemical and Engineering Data, 2006. **51**(5): p. 1668-1671. 10.1021/je060134d.

20. Romdhani, A., et al., *Solubility of sulfacetamide in (ethanol + water) mixtures: Measurement, correlation, thermodynamics, preferential solvation and volumetric contribution at saturation.* Journal of Molecular Liquids, 2019. **290**. 10.1016/j.molliq.2019.111219.

21. Qu, H., et al., *Role of solvent properties and composition on the solid-liquid equilibrium of trifloxystrobin and thermodynamic analysis.* Journal of Molecular Liquids, 2019. **294**. 10.1016/j.molliq.2019.111566.

22. Yu, Z., et al., *Measurement and Correlation of Solubility and Thermodynamic Properties of Vinpocetine in Nine Pure Solvents and (Ethanol + Water) Binary Solvent.* Journal of Chemical and Engineering Data, 2019. **64**(1): p. 150-160. 10.1021/acs.jced.8b00663.

23. Delgado, D.R., et al., *Solution thermodynamics and preferential solvation of sulfamethazine in (methanol + water) mixtures.* Journal of Chemical Thermodynamics, 2016. **97**: p. 264-276. 10.1016/j.jct.2016.02.002.

24. Delgado, D.R. and F. Martínez, *Solubility and solution thermodynamics of sulfamerazine and sulfamethazine in some ethanol+water mixtures.* Fluid Phase Equilibria, 2013. **360**: p. 88-96. 10.1016/j.fluid.2013.09.018.
